# Integrative Multi‐Omics Analysis Identifies Thrombosis‐Associated Molecular Features Linked to Germline Susceptibility and Immune Cell Communication in Gastric Cancer

**DOI:** 10.1111/cbdd.70386

**Published:** 2026-09-01

**Authors:** Xiaogang Lu, Lin Sun, Fujun Jin, Biao Cheng, Ying Huang, Yuzhu Tang, Xiaolong Nie, Feng Gao

**Affiliations:** ^1^ Department of Emergency Xinqiao Hospital, Army Medical University Chongqing China; ^2^ Department of Gastroenterology Chongqing Southeast Hospital Chongqing China; ^3^ Department of Oncology Chongqing Southeast Hospital Chongqing China; ^4^ Department of Oncology Xinqiao Hospital, Army Medical University Chongqing China; ^5^ Department of Radiology Yunyang County Maternal and Child Health Hospital Chongqing China; ^6^ Department of Anesthesiology People’s Hospital of Fengjie County Chongqing China

**Keywords:** ACTN2, CRYAB, gastric cancer, germline susceptibility, thrombosis‐associated molecular signature

## Abstract

Emerging evidence indicates that coagulation‐related molecular programs are associated with thrombosis, tumor progression, and molecular dysregulation in gastric cancer (GC). However, thrombosis‐associated molecular features in GC and their potential links to inherited susceptibility remain insufficiently understood. Integrated analyses of transcriptomic data from The Cancer Genome Atlas (TCGA) and Gene Expression Omnibus (GEO) datasets were performed to identify thrombosis‐associated genes and establish a machine learning‐based prognostic signature. Genome‐wide association study (GWAS), expression quantitative trait loci (eQTL), transcriptome‐wide association study (TWAS), and Mendelian randomization (MR) analyses were conducted to investigate susceptibility‐associated transcriptional programs in GC. Functional assays were used to evaluate candidate genes associated with malignant phenotypes. Single‐cell RNA sequencing (scRNA‐seq) and cell–cell communication analyses were further performed to characterize cell‐type‐specific expression patterns and potential intercellular interactions. A total of 22 differentially expressed thrombosis‐associated genes were identified, and a prognostic signature comprising 14 genes was established. The signature stratified patients into high‐ and low‐risk groups and showed prognostic performance in both the training and validation cohorts. Integrative GWAS, eQTL, and TWAS analyses identified susceptibility‐associated transcriptional programs that were positively correlated with the thrombosis‐associated risk score. Silencing ACTN2 and CRYAB significantly reduced GC cell migration and invasion. scRNA‐seq analysis revealed relatively high CRYAB expression in neutrophils, and CellChat analysis suggested potential neutrophil‐B cell interactions involving COLLAGEN‐related signaling. This integrative multi‐omics study identified a thrombosis‐associated molecular signature linked to prognosis and germline susceptibility‐associated transcriptional programs in GC. ACTN2 and CRYAB may represent candidate genes associated with GC cell migration and invasion, while single‐cell analysis suggested potential immune‐related communication features.

## Graphical abstract

Integrative multi‐omics analysis identified thrombosis‐associated molecular features associated with prognosis and germline susceptibility‐related transcriptional programs in gastric cancer. ACTN2 and CRYAB emerged as candidate molecules linked to gastric cancer cell migration and invasion, with single‐cell analysis suggesting potential neutrophil‐related communication features.

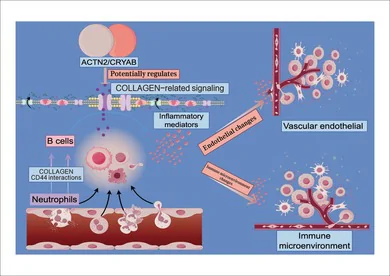

## 1 Introduction

Gastric cancer (GC) remains a major global health burden, accounting for approximately one million new cases and over 650,000 deaths each year worldwide (Sundar et al. 2025). The incidence of GC shows marked geographic variation, with a particularly high burden in East Asia (GBD 2023 Cancer Collaborators 2025). Despite advances in surgery and systemic therapies, the prognosis of GC remains unsatisfactory, partly because many patients are diagnosed at advanced stages and therapeutic resistance remains common (Joshi and Badgwell 2021). Increasing evidence suggests that these clinical challenges are closely related to the complexity of the tumor microenvironment (TME) (Yasuda and Wang 2024). In addition to immune and stromal components, coagulation‐related processes have emerged as important features of tumor biology and may influence tumor progression, immune interactions, and clinical outcomes (Galmiche et al. 2022; Wahab et al. 2023; Monegatti et al. 2025; Aaltonen et al. 2025; Hisada and Mackman 2023).

Cancer‐associated thrombosis is a common manifestation of the procoagulant state in malignancy, and patients with cancer have a substantially elevated risk of thrombotic events compared with the general population (Guntupalli et al. 2023). GC is among the solid tumors with a high thrombotic burden, supporting a close relationship between malignancy and coagulation disorders (Mulder et al. 2021). Beyond clinically evident venous thromboembolism (VTE), coagulation‐related pathways may contribute to tumor growth, angiogenesis, invasion, metastasis, and TME remodeling through mechanisms such as tissue factor overexpression, release of procoagulant molecules, and interactions with immune and stromal cells (Galmiche et al. 2022; Wahab et al. 2023; Mege et al. 2019). Nonetheless, most previous studies have focused primarily on clinically defined thrombotic events and prognosis (Yoshikawa et al. 2023; Song et al. 2025), whereas the molecular characteristics and functional significance of thrombosis‐associated processes in GC remain insufficiently explored.

In this study, we systematically characterized thrombosis‐associated molecular features in GC through integrated multi‐omics analyses. We developed a machine learning–based prognostic signature, examined its potential association with germline susceptibility‐associated transcriptional regulation, and further evaluated key signature genes using functional assays and single‐cell analyses. This integrative framework aimed to provide new insights into the molecular mechanisms linking thrombosis‐associated dysregulation to GC progression.

## 2 Materials and Methods

### 2.1 Data Sources

Transcriptomic profiles and clinical data for GC were obtained from UCSC Xena, including 412 tumor and 36 adjacent normal tissues from The Cancer Genome Atlas Stomach Adenocarcinoma (TCGA‐STAD) cohort. Differential expression analysis was performed using DESeq2, with |log2FC| ≥ 2 and a Benjamini–Hochberg‐adjusted false discovery rate (FDR) < 0.05 used to define differentially expressed genes (DEGs). GSE84437, containing 433 GC samples, was downloaded from the Gene Expression Omnibus (GEO) database as an external validation cohort. The GSE183904 single‐cell RNA sequencing (scRNA‐seq) dataset was obtained from GEO for cell‐type‐specific expression and cell–cell communication analyses. Thrombosis‐associated genes were retrieved from GeneCards using the keyword “thrombosis.” A relevance score‐based screening criterion was applied to reduce the inclusion of weakly associated genes, and 381 thrombosis‐associated genes were retained for subsequent analysis.

### 2.2 Construction and Validation of the Thrombosis‐Associated Risk Model

Differentially expressed thrombosis‐associated genes from TCGA‐STAD were used as candidate features. Expression profiles were log2‐transformed and normalized within each cohort. Because TCGA‐STAD and GSE84437 were used as independent training and validation cohorts rather than merged datasets, no cross‐cohort batch correction was performed. We evaluated 101 machine‐learning algorithm combinations, including Elastic Net, least absolute shrinkage and selection operator (Lasso), Ridge regression, stepwise Cox regression, random survival forest (RSF), CoxBoost, supervised principal components (SuperPC), and survival support vector machine (Survival‐SVM). Model development used 10‐fold cross‐validation, and performance was assessed by the concordance index (C‐index). The optimal model was selected based on performance in both cohorts. Risk scores were calculated using the selected model and interpreted cautiously for cross‐platform validation. Patients were stratified by the cohort‐specific median risk score, and survival differences were assessed using Kaplan–Meier and log‐rank tests.

### 2.3 TWAS, eQTL, and MR Analyses

Transcriptome‐wide association study (TWAS) analysis was performed to examine the relationship between germline susceptibility‐associated transcriptional programs and thrombosis‐related molecular features in GC. GC genome‐wide association study (GWAS) summary statistics were obtained from the IEU OpenGWAS database (ebi‐a‐GCST90018849), and tissue‐specific expression quantitative trait loci (eQTL) reference data were obtained from GTEx v8. TWAS analysis, TWAS‐Score construction, and Mendelian randomization (MR) analyses were conducted using the BioWinford platform (Wang et al. 2026). The TWAS module was based on the FUSION framework, integrating GWAS‐associated single‐nucleotide polymorphism (SNP) signals with eQTL‐mediated transcriptional regulation to evaluate associations between genetically predicted gene expression and GC risk. The TWAS‐Score was calculated from TWAS‐prioritized genes and correlated with the thrombosis‐associated risk score. For selected TWAS‐prioritized genes, two‐sample MR analyses were performed using eligible eQTL variants as instrumental SNP. Associations between genetically predicted gene expression and GC susceptibility were assessed using inverse‐variance weighted (IVW), MR‐Egger, and weighted median methods.

### 2.4 Cell Culture, Transfection, and Functional Assays

HGC‐27 cells were obtained from Wuchuan Biotechnology Co. Ltd. and cultured in RPMI‐1640 medium containing 10% fetal bovine serum and 1% penicillin–streptomycin at 37°C with 5% CO_2_. Small interfering RNAs (siRNAs) targeting ACTN2 and CRYAB were synthesized by Beijing Aoqing Biotechnology Co. Ltd., and transfected according to the manufacturer's instructions. After 72 h, knockdown efficiency was assessed by Western blotting. Total protein was extracted, quantified using a BCA assay kit (B6167, US EVERBRIGHT), separated by SDS‐PAGE, transferred onto PVDF membranes, and incubated with antibodies against ACTN2 (14221‐1‐AP, Proteintech), CRYAB (15808‐1‐AP, Proteintech), and β‐actin (BM0627, BOSTER). Bands were detected using enhanced chemiluminescence (abs920, absin). Migration and invasion were assessed using 8‐μm Transwell inserts, with Matrigel‐coated inserts used for invasion assays. Cells were fixed, stained, and counted in five random fields. All experiments were performed in triplicate.

### 2.5 Functional Enrichment Analysis

Gene Ontology (GO) and Kyoto Encyclopedia of Genes and Genomes (KEGG) analyses were used to evaluate functions and pathways of differentially expressed thrombosis‐associated genes. GO terms were grouped into biological process (BP), cellular component (CC), and molecular function (MF) categories. Results with FDR < 0.05 were considered significant.

### 2.6 Single‐Cell RNA‐Sequencing Analysis

GSE183904 scRNA‐seq data were analyzed using Seurat R package (version 4.0). Cells with < 200 or > 6000 detected genes or > 10% mitochondrial gene expression were excluded. The remaining cells were normalized, scaled, and subjected to highly variable gene selection using FindVariableFeatures, with the top 2000 variable genes retained. Principal component analysis (PCA), graph‐based clustering, and *t*‐distributed stochastic neighbor embedding (t‐SNE) visualization were then performed. Cell types were annotated using canonical marker genes and reference datasets, and ACTN2 and CRYAB expression was examined across cell populations. Cell–cell communication was inferred using CellChat and CellChatDB to identify potential ligand‐receptor interactions and major signaling networks.

### 2.7 Statistical Analysis

All statistical analyses were performed using R software (version 4.2.2). Between‐group differences were assessed using the Wilcoxon rank‐sum test. Correlations were evaluated using Pearson or Spearman analyses, as appropriate. Survival differences were assessed using Kaplan–Meier analysis and log‐rank tests. For functional enrichment analyses, *p* values were adjusted using the Benjamini‐Hochberg method, with an FDR < 0.05 considered significant. Unless otherwise specified, all tests were two‐sided, and *p* < 0.05 was considered significant.

## 3 Results

### 3.1 Identification of Differentially Expressed Thrombosis‐Associated Genes

**FIGURE 1:**
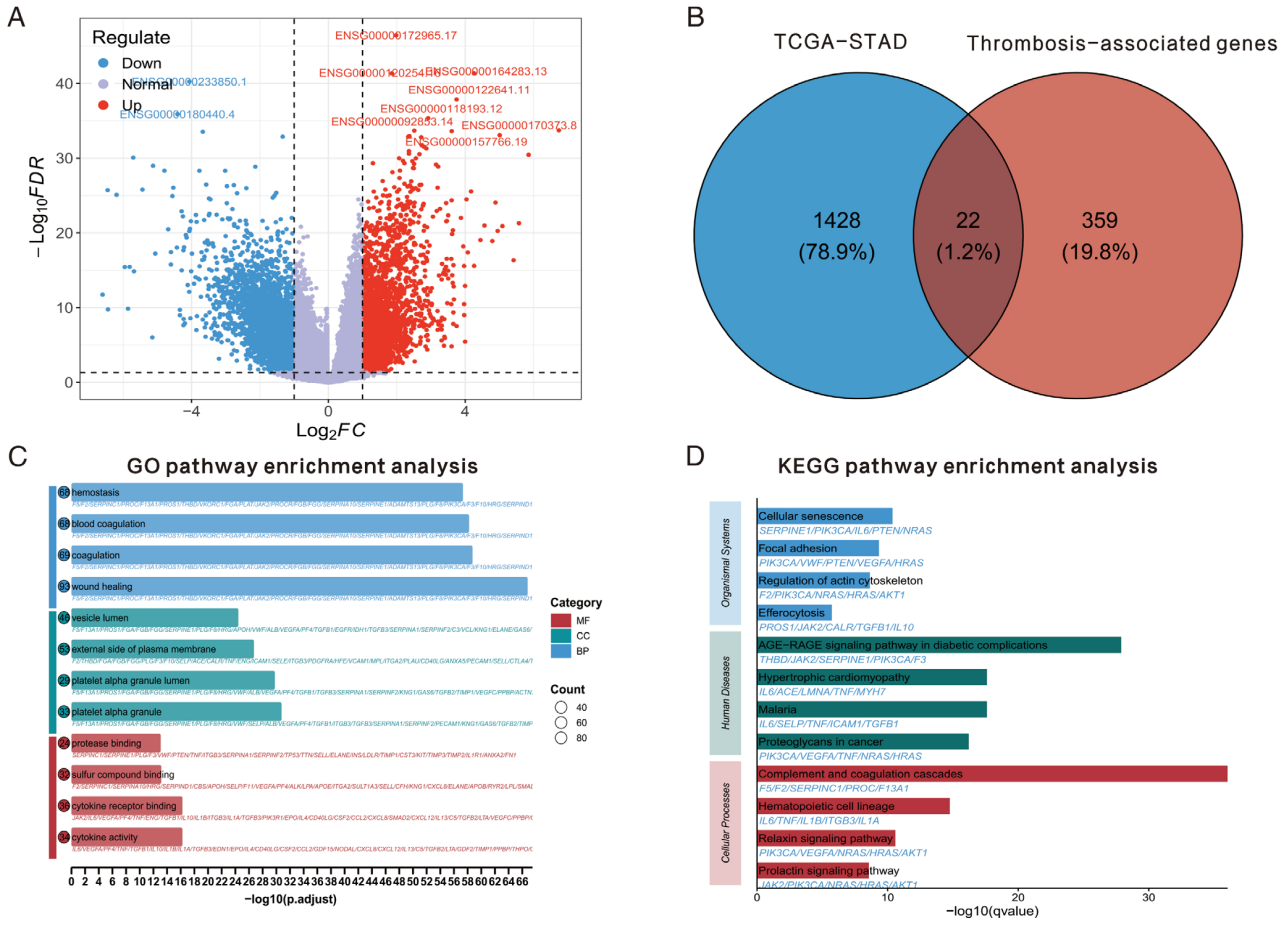
Differentially expressed thrombosis‐associated genes in GC. (A) Volcano plot of DEGs in TCGA‐STAD. (B) Venn diagram of DEGs and thrombosis‐associated genes. (C) GO enrichment analysis. (D) KEGG pathway enrichment analysis.

Differential expression analysis of the TCGA‐STAD cohort identified 1450 DEGs between tumor and normal tissues (Figure 1A). Intersection with 381 GeneCards‐derived thrombosis‐associated genes yielded 22 differentially expressed thrombosis‐associated genes (Figure 1B). Functional enrichment analysis showed that these genes were mainly enriched in coagulation‐related biological processes, including hemostasis, blood coagulation, coagulation, and wound healing. Enriched cellular component terms included vesicle lumen, external side of the plasma membrane, platelet alpha granule lumen, and platelet alpha granule, while molecular function terms included protease binding, sulfur compound binding, cytokine receptor binding, and cytokine activity (Figure 1C). KEGG analysis further indicated enrichment in complement and coagulation cascades, hematopoietic cell lineage, proteoglycans in cancer, focal adhesion, and regulation of actin cytoskeleton pathways (Figure 1D). These differentially expressed thrombosis‐associated genes were then used as candidate features for prognostic model construction.

### 3.2 Construction and Validation of the Thrombosis‐Associated Risk Model

**FIGURE 2:**
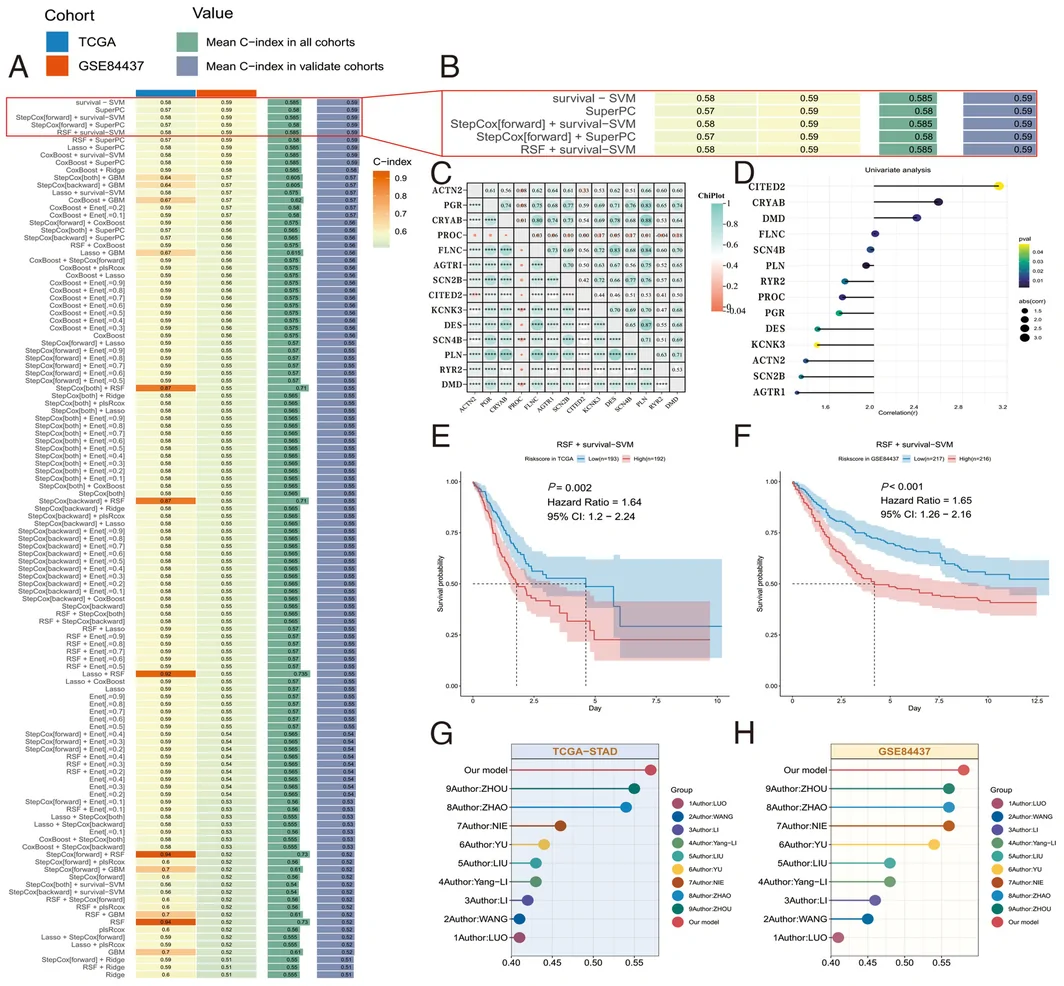
Construction and validation of the thrombosis‐associated risk model. (A) C‐index of machine‐learning models in TCGA‐STAD and GSE84437. (B) Performance of representative models. (C) Correlations among signature genes. (D) Univariate Cox analysis of signature genes. (E, F) Kaplan–Meier survival curves in TCGA‐STAD and GSE84437. (G, H) C‐index comparison with published models in both cohorts.

A prognostic model was constructed using differentially expressed thrombosis‐associated genes in the TCGA‐STAD cohort, with GSE84437 used as an external validation cohort. A total of 101 machine‐learning algorithm combinations were evaluated (Figure 2A). Based on the C‐index, five representative models with stable performance were selected, including Survival‐SVM, SuperPC, StepCox[forward] + Survival‐SVM, StepCox[forward] + SuperPC, and RSF + Survival‐SVM (Figure 2B). Among these models, RSF + Survival‐SVM showed the best overall performance and was selected as the final model, comprising 14 thrombosis‐associated genes. Correlation analysis showed co‐expression patterns among the model genes, and univariate Cox regression indicated associations between several model genes and overall survival in GC patients (Figure 2C,D). Based on the calculated risk scores, patients were stratified into high‐ and low‐risk groups. Kaplan–Meier survival analysis showed that patients in the high‐risk group had poorer overall survival than those in the low‐risk group in both the TCGA‐STAD and GSE84437 cohorts (Figure 2E,F). Comparison with previously published prognostic signatures further showed favorable C‐index performance of the RSF + Survival‐SVM model in both cohorts (Figure 2G,H). These findings support the prognostic relevance of the thrombosis‐associated signature and its further investigation in relation to germline susceptibility‐associated transcriptional programs.

### 3.3 Germline Susceptibility‐Associated Transcriptional Programs Linked to Thrombosis‐Related Molecular Features in GC

**FIGURE 3:**
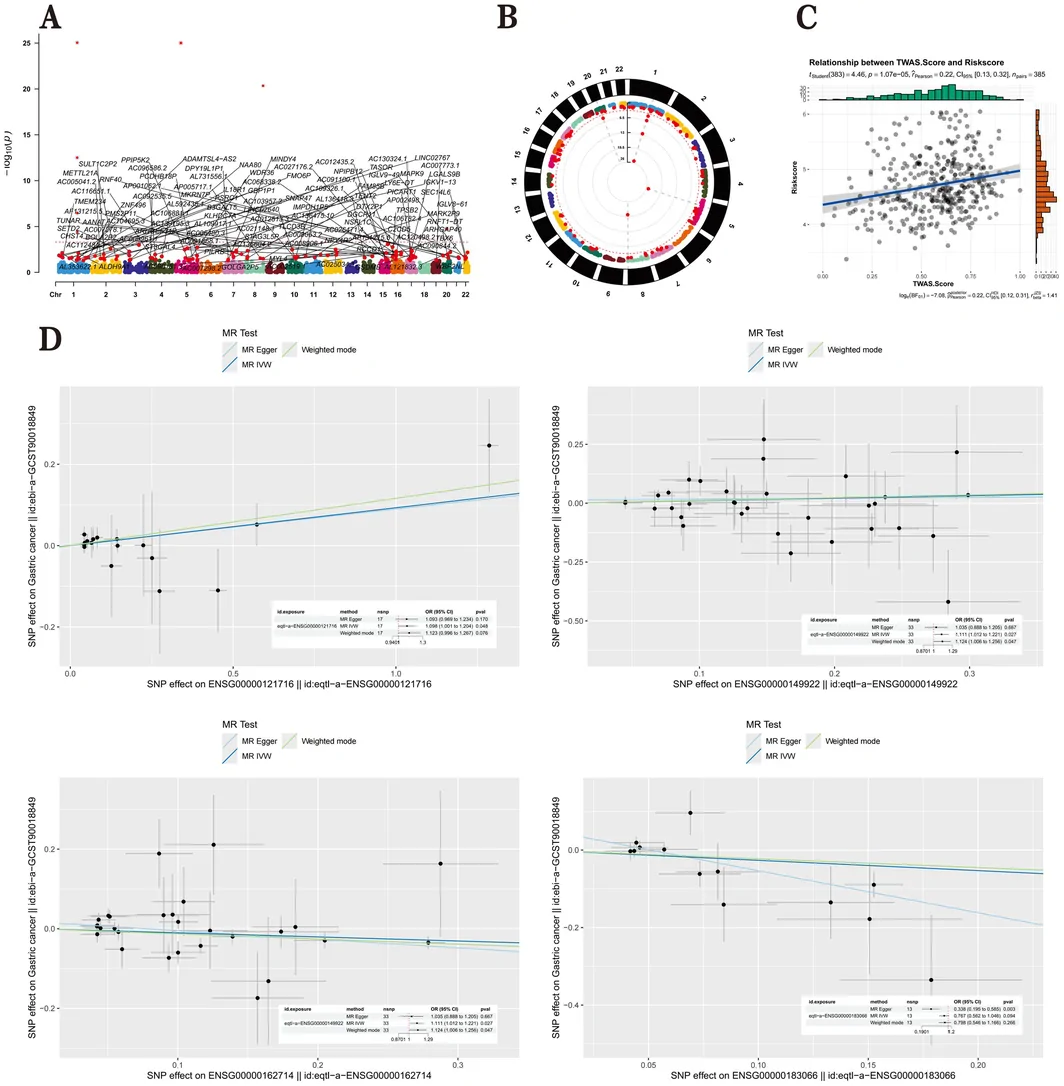
GWAS, TWAS, and MR analyses in GC. (A) Manhattan plot of TWAS results based on GC GWAS summary statistics and eQTL reference data. (B) Chromosomal distribution of TWAS‐positive genes. (C) Correlation between the TWAS‐Score and thrombosis‐associated risk score. (D) MR scatter plots showing potential associations between genetically predicted expression levels of PILRB, TBX6, WBP2NL, and ZNF496 and GC susceptibility.

To explore the potential genetic basis of thrombosis‐related molecular features in GC, TWAS was performed using GC GWAS summary statistics and tissue‐specific eQTL reference datasets. A total of 107 TWAS‐positive candidate genes were identified by integrating GWAS‐associated SNP signals with eQTL‐mediated transcriptional regulation and mapped to multiple genomic regions (Figure 3A,B). The TWAS‐Score derived from these genes was positively correlated with the thrombosis‐associated risk score, suggesting a potential association between germline susceptibility‐associated transcriptional programs and thrombosis‐related molecular features in GC (Figure 3C). Two‐sample MR analyses further provided preliminary genetic evidence that genetically predicted expression levels of PILRB, TBX6, ZNF496, and WBP2NL may be associated with GC susceptibility to varying degrees (Figure 3D). Overall, these results suggest an exploratory link between germline susceptibility‐associated transcriptional programs and thrombosis‐related molecular features in GC rather than direct mechanistic evidence. We next evaluated candidate genes associated with GC cell migration and invasion.

### 3.4 Evaluation of ACTN2 and CRYAB in GC Cell Migration and Invasion

**FIGURE 4:**
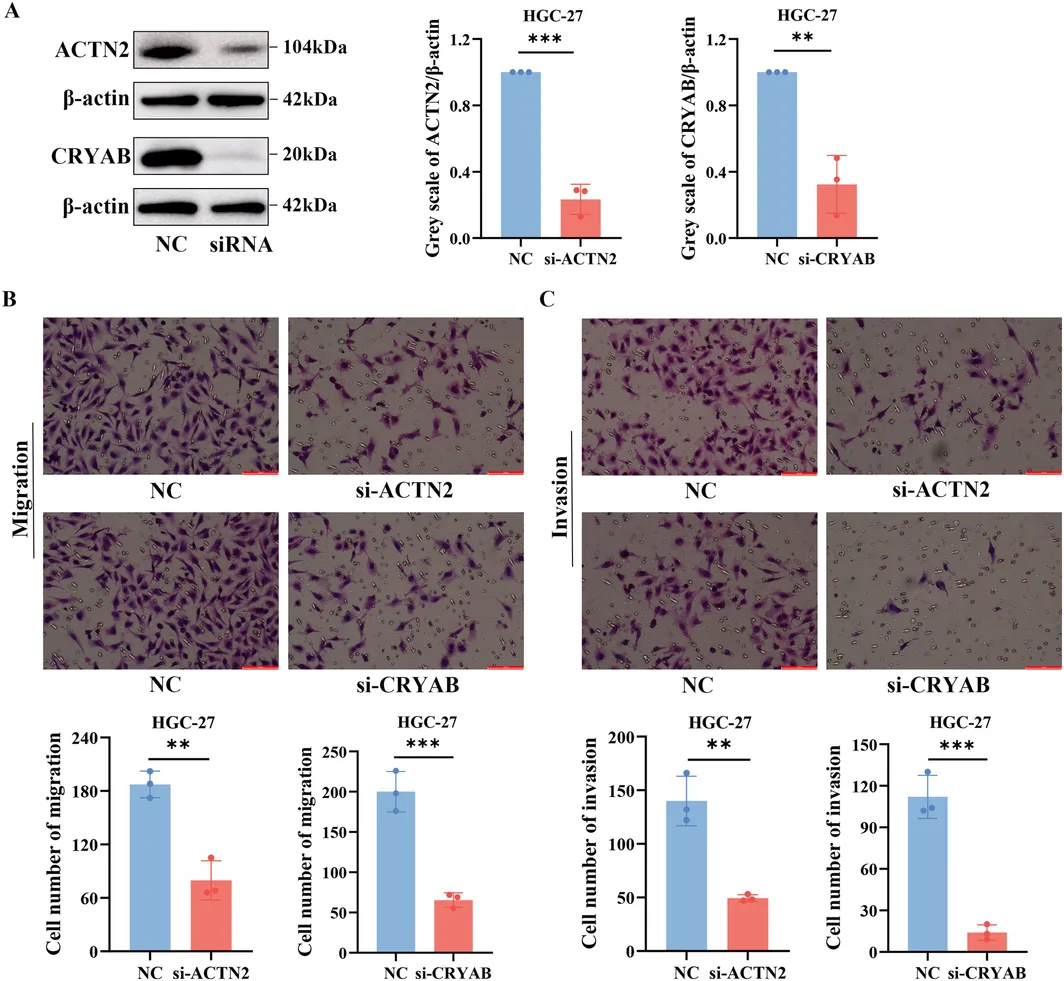
ACTN2 and CRYAB knockdown in HGC‐27 cells. (A) Western blot analysis. (B) Transwell migration assay. (C) Transwell invasion assay. **p < 0.01, ***p < 0.001.

To assess the association of ACTN2 and CRYAB with GC cell migration and invasion, siRNA‐mediated knockdown was performed in HGC‐27 cells. Western blotting confirmed reduced ACTN2 and CRYAB protein levels after transfection (Figure 4A). Transwell assays showed that silencing either gene significantly decreased HGC‐27 cell migration and invasion compared with the negative control group (Figure 4B,C). These findings suggest that ACTN2 and CRYAB are candidate molecules associated with the migratory and invasive phenotypes of HGC‐27 cells. Their cell‐type‐specific expression and potential intercellular communication context were therefore further examined at the single‐cell level.

### 3.5 Single‐Cell Analysis and Cell–Cell Communication of ACTN2 and CRYAB

**FIGURE 5:**
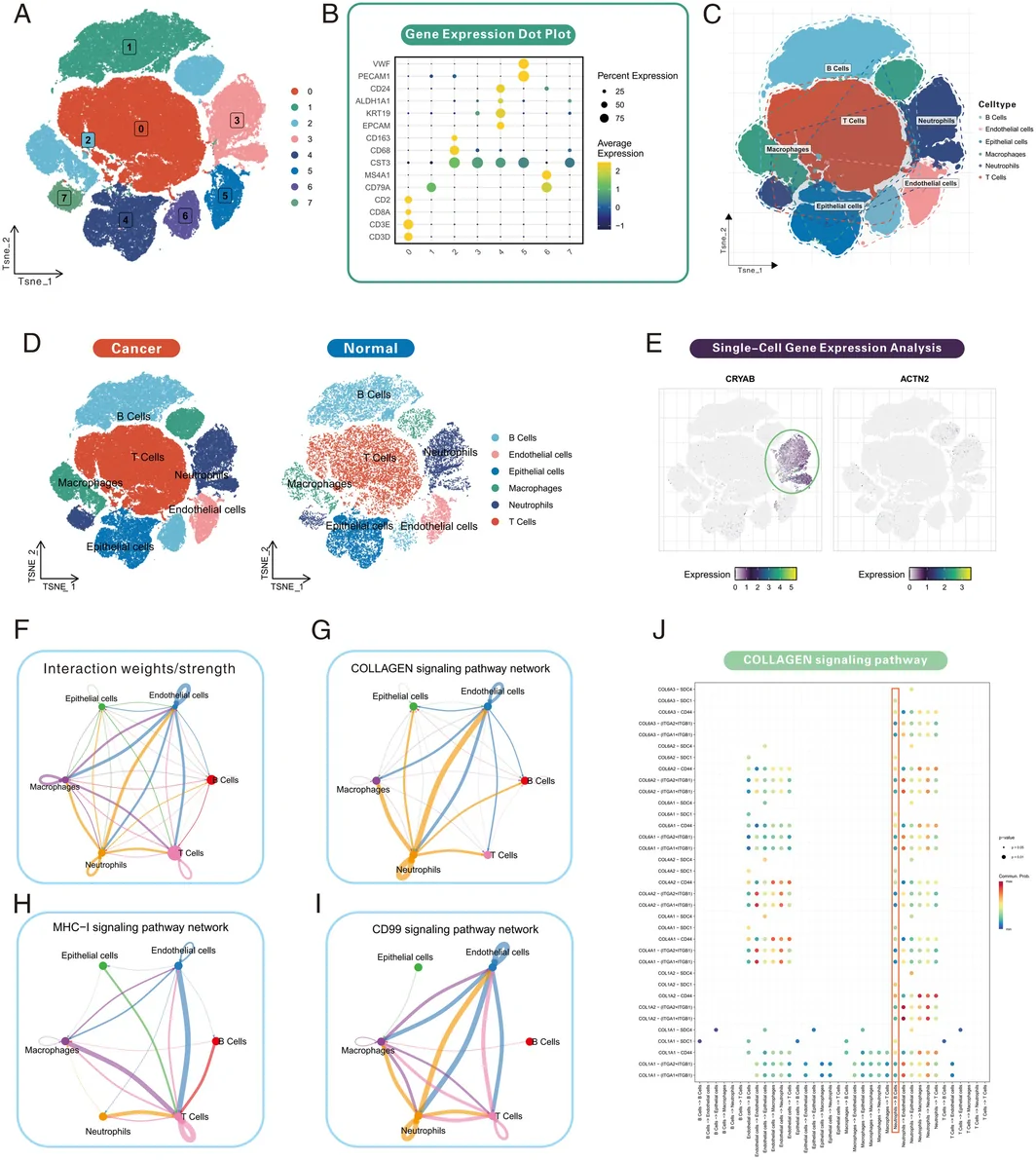
scRNA‐seq and CellChat analyses of ACTN2 and CRYAB. (A) t‐SNE plot of cell clusters. (B) Marker gene expression. (C) Cell‐type annotation. (D) Cell distribution in tumor and normal samples. (E) ACTN2 and CRYAB expression across cell types. (F) Overall CellChat‐inferred communication network. (G–I) CellChat‐inferred COLLAGEN, MHC‐I, and CD99 signaling. (J) Predicted ligand‐receptor pairs in COLLAGEN‐related signaling.

To explore the cellular context of ACTN2 and CRYAB in GC, we analyzed the GSE183904 scRNA‐seq dataset. After normalization and clustering, cells were divided into eight clusters (Figure 5A) and annotated into six major cell types based on canonical markers and the CellMarker2.0 database: epithelial cells, endothelial cells, T cells, B cells, neutrophils, and macrophages (Figure 5B,C). Cell‐type composition differed between tumor and normal tissues (Figure 5D). Across cell types, CRYAB showed relatively higher expression in neutrophils, whereas ACTN2 displayed weaker but detectable expression (Figure 5E). CellChat analysis was then used to infer potential intercellular communication patterns. Predicted interactions were observed between neutrophils and multiple immune cell populations, particularly B cells (Figure 5F). Pathway‐specific analysis identified several inferred signaling networks, including COLLAGEN, MHC‐I, and CD99 (Figure 5G–I), with COLLAGEN‐related signaling showing prominent predicted neutrophil‐B cell interactions. Ligand‐receptor analysis further suggested that CD44 and COL6A3 may act as potential mediators, with neutrophils inferred as signal senders and B cells as receivers (Figure 5J and Figure S1A). ACTN2 and CRYAB were also positively correlated with each other and with CD44 and COL6A3 (Figure S1B–F). Together, these findings suggest a potential cellular context linking ACTN2/CRYAB expression to neutrophil‐associated, CellChat‐inferred communication patterns involving COLLAGEN‐related signaling.

## 4 Discussion

This study systematically characterized thrombosis‐associated molecular features in GC and developed an externally validated prognostic signature. Beyond its prognostic relevance, the risk score showed a potential association with germline susceptibility‐related transcriptional programs, while single‐cell analysis provided clues to immune communication involving neutrophils, B cells, and COLLAGEN‐related signaling. These findings extend previous clinical observations linking VTE to adverse outcomes in GC and suggest that thrombosis‐related molecular changes may also be relevant to antitumor immunity and treatment response (Aaltonen et al. 2025; Yoshikawa et al. 2023; Song et al. 2025; Bahar et al. 2025). Unlike studies focusing mainly on clinically evident thromboembolic events, our analysis highlights thrombosis‐associated molecular dysregulation as a potential layer connecting prognosis, inherited transcriptional signals, and immune microenvironmental features in GC.

Our findings indicate that thrombosis‐associated molecular features in GC are related to both prognosis and germline susceptibility‐associated transcriptional programs. The positive correlation between the TWAS‐Score and thrombosis‐associated risk score suggests a possible association between genetically regulated transcriptional signals and thrombosis‐related molecular alterations, although its biological basis remains unclear. Among the signature genes, ACTN2 and CRYAB were selected for further examination because of their potential biological relevance. ACTN2 has been implicated in cytoskeletal remodeling and cancer cell motility, whereas CRYAB has been linked to cellular stress responses, tumor–stroma interactions, and angiogenesis‐related processes (Lo et al. 2021; Wang et al. 2023; Obacz et al. 2019). Together with the HGC‐27 knockdown results, these findings suggest that ACTN2 and CRYAB are candidate molecules associated with GC cell migration and invasion, while their broader roles in GC progression require further validation.

Single‐cell transcriptomic analysis showed relatively high CRYAB expression in neutrophils, and cell–cell communication analysis suggested possible neutrophil‐B cell interactions, particularly through COLLAGEN‐related signaling networks. This finding is biologically plausible, as neutrophils are increasingly recognized as contributors to tumor progression and cancer‐associated thrombosis through neutrophil extracellular trap formation and other inflammation‐ and coagulation‐related mechanisms (Wang et al. 2024; Ma, Wei, et al. 2024; Li et al. 2023; Yang et al. 2023). In this context, neutrophil‐enriched CRYAB may provide a potential link between thrombosis‐associated molecular features and immune microenvironmental changes in GC. B cells may also contribute to context‐dependent antitumor immune regulation within the TME (Ma, Wu, et al. 2024; Sautès‐Fridman et al. 2019; Schumacher and Thommen 2022; Chen et al. 2025; Wang et al. 2022; Cai et al. 2024). However, the functional relevance of the predicted neutrophil‐B cell interactions remains uncertain, and the COLLAGEN‐related signaling networks identified here were based on computational inference and require further experimental validation.

Several limitations should be acknowledged. Because clinical VTE data were unavailable in the public datasets, the model captures thrombosis‐associated molecular characteristics rather than confirmed thromboembolic events. The associations involving ACTN2, CRYAB, and immune cell interactions were mainly inferred from transcriptomic analyses, CellChat predictions, and HGC‐27 cell experiments, and therefore do not establish causality or support a generalized role in GC progression. Further validation in additional GC cell lines, in vivo models, mechanistic studies, and prospective clinical cohorts is needed. The TWAS‐ and MR‐derived germline susceptibility‐associated signals also require independent validation. In addition, the correlation between the TWAS‐Score and thrombosis‐associated risk score may be influenced by tumor purity, tissue composition, immune infiltration, stromal content, and global expression shifts, and should be interpreted as an exploratory association rather than evidence of a direct mechanistic relationship.

In summary, this study characterized thrombosis‐associated molecular features in GC by integrating transcriptomic profiling, germline susceptibility‐associated analyses, single‐cell transcriptomic analysis, and experimental validation. These features were associated with patient prognosis, genetically regulated transcriptional signals, and immune microenvironment characteristics. Our findings suggest potential links among thrombosis‐associated molecular dysregulation, germline susceptibility‐associated transcriptional programs, and cell–cell communication networks, providing a basis for future validation in GC.

## 5 Conclusion

In conclusion, this study identified a thrombosis‐associated molecular signature linked to prognosis and germline susceptibility‐related transcriptional programs in GC. ACTN2 and CRYAB may represent candidate molecules associated with GC cell migration and invasion, while single‐cell analysis suggested potential immune‐related communication features. These findings support further validation of thrombosis‐associated molecular dysregulation in GC.

## Author Contributions

**Xiaogang Lu:** conceptualization, methodology, investigation, validation, formal analysis, data curation, software, writing – original draft, writing – review and editing. **Lin Sun:** investigation, methodology, validation, formal analysis, data curation, visualization, writing – original draft, writing – review and editing. **Fujun Jin:** investigation, validation, formal analysis, data curation, writing – original draft. **Biao Cheng:** formal analysis, data curation, visualization. **Ying Huang:** investigation, validation. **Feng Gao:** conceptualization, methodology, supervision, project administration, writing – review and editing. **Yuzhu Tang:** data curation. **Xiaolong Nie:** data curation.

## Funding

The authors have nothing to report.

## Conflicts of Interest

The authors declare no conflicts of interest.

## Supporting information


**Figure S1:** Association between ACTN2, CRYAB, and COLLAGEN‐related signaling. (A) Overview of COLLAGEN‐related signaling interactions across different cell types. (B–F) Correlation analyses between ACTN2, CRYAB, and key genes involved in COLLAGEN‐related signaling (including COL6A3 and CD44). Correlation coefficients (*r*) and corresponding *p* values are shown.

## Data Availability

The datasets analyzed in this study are publicly available. Transcriptomic and clinical data for gastric cancer (GC) were obtained from the UCSC Xena platform using The Cancer Genome Atlas Stomach Adenocarcinoma (TCGA‐STAD) cohort. The GSE84437 and GSE183904 datasets were downloaded from the Gene Expression Omnibus (GEO) database. Genome‐wide association study (GWAS) summary statistics were obtained from the IEU OpenGWAS database, including dataset ebi‐a‐GCST90018849. Tissue‐specific expression quantitative trait loci (eQTL) reference data were obtained from the Genotype‐Tissue Expression (GTEx) project. Transcriptome‐wide association study (TWAS) and Mendelian randomization (MR) analyses were performed using the BioWinford platform. All datasets used in this study are publicly accessible. Additional information supporting the findings of this study is available from the corresponding author upon reasonable request.

## References

[cbdd70386-bib-0001] Aaltonen, P. , H. Mustonen , P. Puolakkainen , C. Haglund , K. Peltola , and H. Seppänen . 2025. “Incidence and Prognostic Role of Venous Thromboembolism in Gastric Cancer: A Nationwide Finnish Register Study.” European Journal of Surgical Oncology 51: 110139. 10.1016/j.ejso.2025.110139. 40373555

[cbdd70386-bib-0002] Bahar, F. , B. Ibis , S. Cakir Colak , et al. 2025. “Cardiovascular and Thromboembolic Outcomes With Immune Checkpoint Inhibitors in Gastroesophageal Cancer: A Propensity Score‐Matched Cohort Study.” Gastric Cancer 28: 550–555. 10.1007/s10120-025-01582-1. 39838247

[cbdd70386-bib-0003] Cai, X. , J. Yang , Y. Guo , Y. Yu , C. Zheng , and X. Dai . 2024. “Re‐Analysis of Single Cell and Spatial Transcriptomics Data Reveals B Cell Landscape in Gastric Cancer Microenvironment and Its Potential Crosstalk With Tumor Cells for Clinical Prognosis.” Journal of Translational Medicine 22: 807. 10.1186/s12967-024-05606-9. 39215354 PMC11365245

[cbdd70386-bib-0004] Chen, W. , L. Zhang , M. Gao , et al. 2025. “Role of Tertiary Lymphoid Structures and B Cells in Clinical Immunotherapy of Gastric Cancer.” Frontiers in Immunology 15: 1519034. 10.3389/fimmu.2024.1519034. 39840050 PMC11747648

[cbdd70386-bib-0005] Galmiche, A. , J. Rak , L. T. Roumenina , and Z. Saidak . 2022. “Coagulome and the Tumor Microenvironment: An Actionable Interplay.” Trends in Cancer 8: 369–383. 10.1016/j.trecan.2021.12.008. 35027336

[cbdd70386-bib-0006] GBD 2023 Cancer Collaborators . 2025. “The Global, Regional, and National Burden of Cancer, 1990–2023, With Forecasts to 2050: A Systematic Analysis for the Global Burden of Disease Study 2023.” Lancet 406: 1565–1586. 10.1016/S0140-6736(25)01635-6. 41015051 PMC12687902

[cbdd70386-bib-0007] Guntupalli, S. R. , D. Spinosa , S. Wethington , R. Eskander , and A. A. Khorana . 2023. “Prevention of Venous Thromboembolism in Patients With Cancer.” BMJ 381: e072715. 10.1136/bmj-2022-072715. 37263632

[cbdd70386-bib-0008] Hisada, Y. , and N. Mackman . 2023. “Mechanisms of Cancer‐Associated Thrombosis.” Research and Practice in Thrombosis and Haemostasis 7: 100123. 10.1016/j.rpth.2023.100123. 37122533 PMC10139954

[cbdd70386-bib-0009] Joshi, S. S. , and B. D. Badgwell . 2021. “Current Treatment and Recent Progress in Gastric Cancer.” CA: A Cancer Journal for Clinicians 71: 264–279. 10.3322/caac.21657. 33592120 PMC9927927

[cbdd70386-bib-0010] Li, J. , Y. Xia , B. Sun , et al. 2023. “Neutrophil Extracellular Traps Induced by the Hypoxic Microenvironment in Gastric Cancer Augment Tumour Growth.” Cell Communication and Signaling 21: 86. 10.1186/s12964-023-01112-5. 37127629 PMC10152773

[cbdd70386-bib-0011] Lo, L. H. , C. Y. Lam , J. C. To , C. H. Chiu , and V. W. Keng . 2021. “Sleeping Beauty Insertional Mutagenesis Screen Identifies the Pro‐Metastatic Roles of CNPY2 and ACTN2 in Hepatocellular Carcinoma Tumor Progression.” Biochemical and Biophysical Research Communications 541: 70–77. 10.1016/j.bbrc.2021.01.017. 33482578

[cbdd70386-bib-0012] Ma, J. , Y. Wu , L. Ma , et al. 2024. “A Blueprint for Tumor‐Infiltrating B Cells Across Human Cancers.” Science 384: eadj4857. 10.1126/science.adj4857. 38696569

[cbdd70386-bib-0013] Ma, Y. , J. Wei , W. He , and J. Ren . 2024. “Neutrophil Extracellular Traps in Cancer.” MedComm 5: e647. 10.1002/mco2.647. 39015554 PMC11247337

[cbdd70386-bib-0014] Mege, D. , L. Panicot‐Dubois , and C. Dubois . 2019. “Mechanisms of Cancer‐Associated Thrombosis.” HemaSphere 3: 19–21. 10.1097/HS9.0000000000000239. 35309769 PMC8925691

[cbdd70386-bib-0015] Monegatti, S. , N. Martinelli , S. Friso , H. M. H. Spronk , and H. Ten Cate . 2025. “Mechanisms and Management of Thrombosis in Cancer: Focus on Gastrointestinal Malignancies.” Journal of Pharmacology and Experimental Therapeutics 392: 100018. 10.1124/jpet.124.002203. 39893001

[cbdd70386-bib-0016] Mulder, F. I. , E. Horváth‐Puhó , N. van Es , et al. 2021. “Venous Thromboembolism in Cancer Patients: A Population‐Based Cohort Study.” Blood 137: 1959–1969. 10.1182/blood.2020007338. 33171494

[cbdd70386-bib-0017] Obacz, J. , T. Avril , C. Rubio‐Patiño , et al. 2019. “Regulation of Tumor‐Stroma Interactions by the Unfolded Protein Response.” FEBS Journal 286: 279–296. 10.1111/febs.14359. 29239107

[cbdd70386-bib-0018] Sautès‐Fridman, C. , F. Petitprez , J. Calderaro , and W. H. Fridman . 2019. “Tertiary Lymphoid Structures in the Era of Cancer Immunotherapy.” Nature Reviews Cancer 19: 307–325. 10.1038/s41568-019-0144-6. 31092904

[cbdd70386-bib-0019] Schumacher, T. N. , and D. S. Thommen . 2022. “Tertiary Lymphoid Structures in Cancer.” Science 375: eabf9419. 10.1126/science.abf9419. 34990248

[cbdd70386-bib-0020] Song, J. , A. A. Morgan , J. Ahn , et al. 2025. “Validating Khorana Risk Score in Gastric Cancer Patients on Immune Checkpoint Inhibitors and Chemotherapy.” Immunotherapy 17: 419–424. 10.1080/1750743X.2025.2501922. 40336190 PMC12091900

[cbdd70386-bib-0021] Sundar, R. , I. Nakayama , S. R. Markar , et al. 2025. “Gastric Cancer.” Lancet 405: 2087–2102. 10.1016/S0140-6736(25)00052-2. 40319897

[cbdd70386-bib-0022] Wahab, R. , M. M. Hasan , Z. Azam , P. J. Grippo , and T. A. Al‐Hilal . 2023. “The Role of Coagulome in the Tumor Immune Microenvironment.” Advanced Drug Delivery Reviews 200: 115027. 10.1016/j.addr.2023.115027. 37517779 PMC11099942

[cbdd70386-bib-0023] Wang, C. , B. Xie , S. Yin , et al. 2023. “Induction of Filopodia Formation by α‐Actinin‐2 via RelA With a Feedforward Activation Loop Promoting Overt Bone Marrow Metastasis of Gastric Cancer.” Journal of Translational Medicine 21: 399. 10.1186/s12967-023-04156-w. 37337244 PMC10280853

[cbdd70386-bib-0024] Wang, H. , S. J. Kim , Y. Lei , et al. 2024. “Neutrophil Extracellular Traps in Homeostasis and Disease.” Signal Transduction and Targeted Therapy 9: 235. 10.1038/s41392-024-01933-x. 39300084 PMC11415080

[cbdd70386-bib-0025] Wang, Y. , T. Wu , X. Lu , X. Wang , and W. Xue . 2026. “BioWinfordMR: An Online Platform for Comprehensive Mendelian Randomization Analysis.” Frontiers in Immunology 16: 1695146. 10.3389/fimmu.2025.1695146. 41607796 PMC12835390

[cbdd70386-bib-0026] Wang, Z. , Z. Lu , S. Lin , et al. 2022. “Leucine‐tRNA‐Synthase‐2‐Expressing B Cells Contribute to Colorectal Cancer Immunoevasion.” Immunity 55: 1067–1081.e8. 10.1016/j.immuni.2022.04.017. 35659337

[cbdd70386-bib-0027] Yang, S. , B. Sun , J. Li , et al. 2023. “Neutrophil Extracellular Traps Promote Angiogenesis in Gastric Cancer.” Cell Communication and Signaling 21: 176. 10.1186/s12964-023-01196-z. 37480055 PMC10362668

[cbdd70386-bib-0028] Yasuda, T. , and Y. A. Wang . 2024. “Gastric Cancer Immunosuppressive Microenvironment Heterogeneity: Implications for Therapy Development.” Trends in Cancer 10: 627–642. 10.1016/j.trecan.2024.03.008. 38600020 PMC11292672

[cbdd70386-bib-0029] Yoshikawa, T. , T. Sano , M. Terashima , et al. 2023. “Incidence and Risk Factors for Venous Thromboembolism in the Cancer‐VTE Registry Stomach Cancer Subcohort.” Gastric Cancer 26: 493–503. 10.1007/s10120-023-01378-1. 37004667 PMC10284943

