# Supplementary material for: Integrative Multi‐Omics Analysis Identifies Thrombosis‐Associated Molecular Features Linked to Germline Susceptibility and Immune Cell Communication in Gastric Cancer
Source: Chem Biol Drug Des. 2026 Sep 1;108(3):e70386. doi: 10.1111/cbdd.70386 (PMC13534880; doi:10.1111/cbdd.70386)
Supplement: Supplementary file 1 [file CBDD-108-e70386-s001.zip › Supplementary_Materials_revised_with_corrected_Figure4A_WB_data/Supplementary figure.docx]

**Supplementary Figures**

**Supplementary Figure 1. Association between ACTN2, CRYAB, and COLLAGEN-related signaling.**

(A) Overview of COLLAGEN-related signaling interactions across different cell types. (B–F) Correlation analyses between ACTN2, CRYAB, and key genes involved in COLLAGEN-related signaling (including COL6A3 and CD44). Correlation coefficients (r) and corresponding *p* values are shown.
