# Supplementary material for: Integrative Multi‐Omics Analysis Identifies Thrombosis‐Associated Molecular Features Linked to Germline Susceptibility and Immune Cell Communication in Gastric Cancer
Source: Chem Biol Drug Des. 2026 Sep 1;108(3):e70386. doi: 10.1111/cbdd.70386 (PMC13534880; doi:10.1111/cbdd.70386)
Supplement: Supplementary file 1 [file CBDD-108-e70386-s001.zip › Supplementary_Materials_revised_with_corrected_Figure4A_WB_data/Supplementary File 2-WB-all data/wb-CRYAB.pdf]

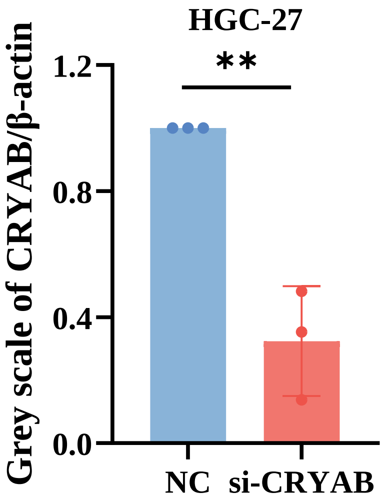

HGC-27
**
Grey scale of CRYAB/β-actin
1.2
0.8
0.4
0.0
NC
si-CRYAB
